# Early-life neural correlates of behavioral inhibition and anxiety risk

**DOI:** 10.1038/s41386-025-02235-8

**Published:** 2025-10-04

**Authors:** Courtney A. Filippi, Alice Massera, Jiayin Xing, Luis Martinez Agulleiro

**Affiliations:** Department of Child and Adolescent Psychiatry, New York University, New York, NY USA

**Keywords:** Human behaviour, Risk factors

## Abstract

This review showcases the ways that studying the neural basis of Behavioral Inhibition (BI) and maternal anxiety in infancy has advanced our understanding of the developmental pathophysiology of anxiety. We demonstrate that infants with BI and those who have been exposed to maternal anxiety/stress exhibit differences in neural processes associated with bottom-up attention and top-down control, both when we measure the brain at rest and when we measure the brain during stimulus processing. Differences in infant stimulus processing are particularly robust—not only do they emerge in at-risk infants, but they also shape risk trajectories from infancy through adolescence. Throughout this review, we underscore the value in a focus on infancy and early childhood. We also point to several key future directions for this work, including prioritizing a longitudinal, multi-modal approach for studying neurobehavioral profiles of early-life risk. Together, this work demonstrates that neural processes involved in attention and control are central to BI and early-life risk for anxiety across the lifespan.

## Introduction

Anxiety disorders are among the most prevalent psychiatric illnesses in children [1] and emerge remarkably early—with some children exhibiting impairing anxiety as early as preschool. When left untreated, anxiety can profoundly impact a child’s developmental trajectory. Despite decades of research on childhood anxiety illuminating several promising markers of risk, our ability to pinpoint which individuals are likely to develop anxiety remains limited. This gap in our understanding of the mechanisms that underlie the development of anxiety impacts early identification and intervention efforts. This review underscores the ways in which a developmental neuroscience lens focused on the period between infancy and preschool has led to promising advancements in the developmental pathophysiology of anxiety. To do so, we situate our discussion in one of the most robust behavioral markers of early-life risk: behavioral inhibition (BI), an infant temperament characterized by novelty-evoked distress and avoidance. BI serves as the behavioral anchor throughout this review; largely constraining our discussion to those elements of the temperament literature that characterize novelty-evoked expressions fear or distress in infancy rather than all negative emotions (see Box 1). We suggest that being specific about the context in which these behaviors emerge may support efforts to delineate trajectories of risk. In doing so, we also acknowledge that BI does not emerge in isolation; BI and anxiety are moderately heritable [2–5] and have been linked to maternal factors (e.g., elevated prenatal stress and maternal anxiety [3, 6–10]), which likely reciprocally impact BI. Thus, this review is inclusive of these factors.

To begin, we review infant behavioral risk phenotypes of anxiety and the prevailing neuroscientific model of anxiety disorders. Next, we illustrate how infancy research has examined the neural correlates of these risk phenotypes and identified neural mechanisms that mediate and moderate trajectories from risk phenotypes to anxiety symptoms. In doing so, we highlight the ways in which longitudinal data has illuminated gaps in existing neurodevelopmental models of anxiety. We also demonstrate that existing work has harnessed the variability, both across children and across time, that is inherent to change during this period; an approach in line with decades of developmental science research [11, 12]. Such work, which fundamentally targets trajectories of change, is needed to further refine neurodevelopmental models of anxiety. Overall, we argue that infant neuroimaging studies provide promising evidence that differences in neural processing supporting attentional control may underlie the development of anxiety.

Throughout this review, we additionally underscore that infancy is particularly valuable for studying risk for anxiety because it is a sensitive period for social-emotional development and learning about one’s environment. During infancy, the attentional system becomes preferentially tuned to stimuli [13, 14], and this learning can have a lifelong impact on perceptual systems [15, 16]. Infancy is also characterized by profound brain changes that impact all foundational cognitive systems [17, 18]. It is (arguably) among the best developmental stages for detecting biologically (or genetically) embedded constraints on neural/cognitive systems [19, 20]. We posit that the study of developmental pathophysiology has and will continue to benefit enormously from these features of this developmental stage.

Box 1 Behavioral Inhibition (BI) and Negative Reactivity (NR) were originally defined by Jerome Kagan as behavioral reactions that are unique to the context of novelty. While many temperament classifications exist, they do not all define behavior in context in the way that Kagan did. Characterizing the context that elicits distress, and avoidance is among the chief strengths of observational assessments. Even so, several highly similar temperament constructs are related to NR and BI. Highly similar terms include: dysregulated fear and negative emotionality/negative affectivity. *Dysregulated fear* refers to a fear response that the child struggles to regulate or manage. It is distinguished by being persistent especially in non-threatening contexts. *Negative emotionality/affectivity* refers to the tendency for a child to experience negative emotions (e.g., sadness, distress, anger). While negative emotionality is correlated with BI, negative emotionality does not capture fear of novelty specifically. Thus, in anchoring this review in BI, we do not focus on findings associated with negative emotionality (although see Table 2 for comparison).

## Behavioral risk phenotypes and anxiety

**Fig. 1:**
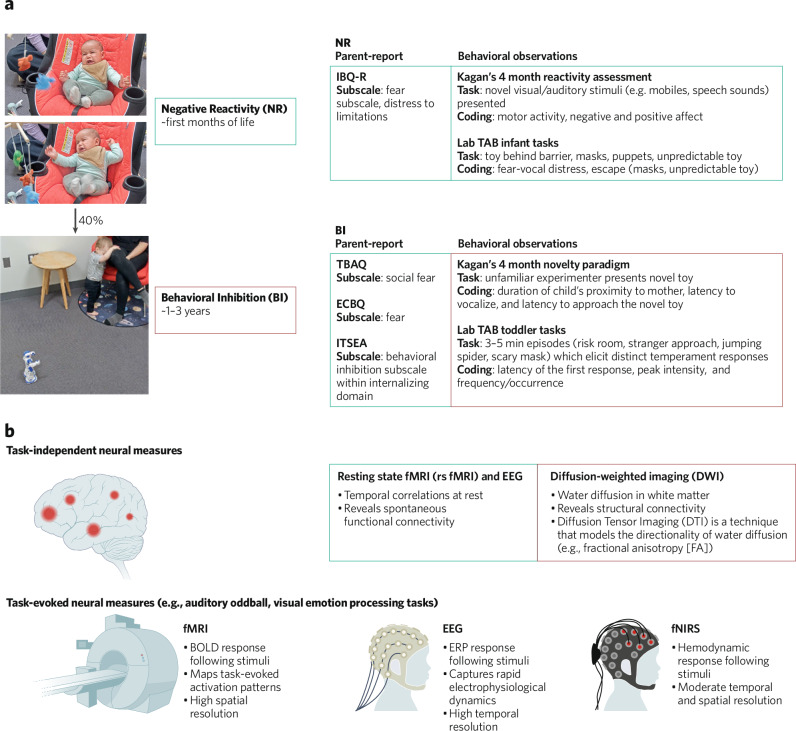
Negative Reactivity (NR), Behavioral Inhibition (BI), and Neuroscience tools used to study neural correlates of these behaviors. **a** Illustrates NR and BI and provides common examples of how these constructs are measured using parent-report and behavioral observation. **b** Overview of relevant imaging measures. Authors affirm that they obtained informed consent for publication of the images.

One of the best understood behavioral markers of early-life risk for anxiety is BI, a temperament characterized by distress and avoidance of novelty in toddlerhood [21]. BI is relatively stable across time, and is commonly measured using observational assessments (i.e., in standardized laboratory tasks) or parent reports [22–24]. See Fig. 1 and Box 1. Children with BI are estimated to be four times more likely to develop anxiety (often social anxiety) than children without this temperament [25, 26]. However, not all children with BI develop anxiety disorders. Approximately 40% of BI children develop an anxiety disorder later in life [25]. Despite the heterogeneity in anxiety outcomes of behaviorally inhibited children, we know that BI has an enduring impact on social-emotional outcomes more broadly. To illustrate, even behaviorally inhibited children who do *not* ultimately receive an anxiety diagnosis struggle with hyper-responsivity to novelty [27], social rejection, and peer interactions [28]. As adults, those with a history of BI exhibit more social withdrawal (i.e., avoidance of social situations), loneliness, and are less interpersonally effective [29].

While BI is the most widely studied early-life risk marker for anxiety, individual differences in infants’ responses to novelty can be detected before toddlerhood. Negative reactivity (NR), a behavioral profile characterized by novelty-evoked distress (see Fig. 1), emerges at 4 months of age and often precedes BI [30–32]. While NR, BI, and anxiety are independent constructs, the similarities in behavioral profiles have led to the hypothesis that anxiety disorders may, in part, have developmental origins in these early-life temperaments (i.e., NR and BI). This idea, while supported to some extent, is embedded in complexity [33]. Some of this complexity is developmental in nature—that is to say, 4-month-olds and 2-year-olds do not have the behavioral repertoire required to meet criteria for an anxiety disorder. This aspect of the problem makes it difficult to tease apart the nature of the relation between these temperaments and anxiety. Rather than seeking to understand which is the cause or consequence, we suggest that this literature is best approached from the perspective that these phenotypes likely indicate some degree of shared underlying neurobiological mechanisms. From this standpoint, we consider these “risk phenotypes” (i.e., NR, BI) as valuable because they provide clues about neural systems that are contributing to relevant patterns of fearful and anxious behavior.

Both NR and BI are associated with atypical attentional processing; most commonly, aberrant salience and threat processing [34]. Children with BI struggle to deploy attention under stress [35]. By adolescence, children with BI preferentially attend to threat cues [36] and exhibit heightened attention to their own errors [37–39]. Building on studies of anxious patients, it has been hypothesized that anxiety and early-life behavioral risk phenotypes emerge due to an imbalance of stimulus-driven (“bottom-up”) attention to threat and cortically mediated (“top-down”) behavioral control processes [40, 41]. This imbalance has been linked to differences in several attention-related brain networks, such as the salience network (SN), fronto-parietal network (FPN), dorsal attention network (DAN) and default mode network (DMN). Considerable evidence from adults and children supports this theory [42–52], as does work in animal models with high degrees of experimental control and direct causal manipulations [53–59]–suggesting evolutionary conservation of the processes that underlie fear and anxiety. Even so, this hypothesis has only recently begun to be tested in human infants. In the sections that follow, we review these studies.

## The infant brain at rest

This section reviews advancements in our understanding of the neural correlates of BI derived from studying the infant brain at rest. To date, two neuroscientific methods have been used to do so: magnetic resonance imaging (MRI) and electroencephalography (EEG). MRI provides data with high spatial resolution facilitating the mapping of synchronized or correlated patterns of neural activity across large-scale networks (i.e., functional connectivity) and provides data on white matter microstructure (i.e., structural connectivity) in sleeping infants. In contrast, EEG provides data with high temporal resolution (albeit limited spatial resolution) to provide information about coordinated electrical activity across the scalp in awake infants (see also [60]). This section reviews how findings across these two modalities are shaping our understanding of how task-independent processing relates to early-life risk.

### MRI: functional and structural connectivity

Resting state functional magnetic resonance imaging (rs-fMRI) evaluates how brain activity is synchronized across different brain regions when the brain is not performing a specific task [61]. This method has shown that, on average, adults’ brains organize into large-scale networks, including the brain networks supporting the dynamic control of attention highlighted above (i.e., SN, FPN, DAN, DMN; [62]). Resting state functional connectivity in infants is largely similar to that of adults [63–65]. While there are some exceptions (e.g., DMN), this suggests reasonable developmental stability in brain network organization.

#### Cross-sectional rs-fMRI

Across both fMRI studies of infants and adults, the individual variability in the strength of the connections within and between different networks relates to individual differences in fearful behavior. Adults and children with anxiety disorders exhibit resting state functional connectivity (rs-FC) differences in the DAN [66], SN [66–69], FPN [66, 68, 70], and DMN [66, 68]. Individual differences in infant BI and NR have also been associated with individual differences in rs-FC in these same attention-related networks. Specifically, infant fMRI studies find that heightened within-SN connectivity (e.g., amygdala–insula, amygdala–cingulate connections) is associated with greater NR [71–73], BI [73], and internalizing symptoms (at age 2; [74]; see Table 1 for summary of findings). Reduced infant DMN connectivity [75, 76] is associated with greater BI. Additionally, weaker DAN–DMN, DAN–SN, and DAN–FPN connectivity is associated with greater NR in infancy [77].

**Table 1:** Summary of selected studies.

Author (year)	*N*	Age at imaging	Temperament/psychopathology dimension	Temperament/psychopathology measure	Brain measure	Main findings
Task-based EEG						
Bowman et al. [158]	142	5–12 months	Maternal anxiety	STAI	Emotional processing (visual stimuli) EEG (awake): NC, N290, P450	Greater maternal anxiety predicted more negative NC amplitude for happy and fearful faces in left- and mid-central scalp regions.
Marshall et al. [142]	103	9 months	NR	Observation in the laboratory (Kagan reactivity assessment)	Novelty processing (auditory stimuli) EEG (awake): P3	High negative reactive infants show an increase amplitude of a slow wave in the P3 response to deviant tones. High positive reactive infants show a larger P3 to novel tones.
Otte et al. [157]	82	9–11 months	Maternal anxiety	STAI	Emotional processing (auditory and visual stimuli) EEG (awake): P150, N450, P350	High maternal anxiety is associated with high infant P350 response to fearful vocalizations (and low for happy vocalizations).
Rayson et al. [167]	71	3–5 years	BI Child anxiety	BIQ Observed assessment: Lab TAB Preschool Anxiety Scale	Emotional processing (visual stimuli) EEG (awake): P1, P2, N2	BI positively predicted anxiety symptoms. BI is associated with higher anxiety when an angry bias on N2 is present. BI is associated with higher anxiety when a happy bias on P1 is present.
Task-evoked fMRI						
Schwarzlose et al. [143]	27	1–13 months	BI	Observation in laboratory (Kagan BI assessment)	Novelty processing (auditory oddball paradigm) T-fMRI (asleep)	Increased activation (at age 1 month) during deviant stimuli in PFC and subcortical regions (including amygdala) are positively associated with BI score at age 1 year.
Sylvester et al. [144]	45	1 month	Maternal anxiety	STAI	Novelty processing (auditory oddball paradigm) T-fMRI (asleep)	Deviant stimuli increased brain activity in brain areas in a similar pattern as adults (i.e., dACC, insula). Maternal anxiety is associated with increased response to deviant sounds in insula, vlPFC and cingulate.
Resting state fMRI						
Dufford et al. [78]	20	24 months	Maternal anxiety	STAI	R-fMRI (asleep)	Greater maternal trait anxiety was associated with reduced amygdala-ACC functional connectivity.
Filippi et al. [71]	34	4 months	NR	Observation in laboratory (Kagan reactivity assessment) IBQ	R-fMRI (asleep)	Greater amygdala cingulate and amygdala-superior frontal gyrus connectivity was associated with low positive affect. Amygdala-cingulate connectivity was associated with greater negative affect compared to positive affect.
Filippi et al. [77]	101	4 months	NR	IBQ and Observation in laboratory (Kagan reactivity assessment)	R-fMRI (asleep)	Parent-reported NR was associated with less DAN-DMN, DAN-SN, DAN-FPN connectivity. Observed NR was associated with less DAN-FPN connectivity.
Filippi et al. [99]	180	0–60 months	BI	IBQ, ECBQ	R-fMRI (asleep)	Smaller declines in FC between DAN and FPN over time are associated with greater fearful temperament at age 2 years. Low initial FC between DAN and FPN is associated with increased fearfulness at age 2 years.
Graham et al. [72]	48	1 month	Fear	IBQ	R-fMRI (asleep)	Greater amygdala-cingulate/medial prefrontal cortex connectivity was associated with high fear and advanced cognitive development.
Marr et al. [80]	60	1–3 months	Maternal perinatal stress	Center for Epidemiologic Studies Depression Scale, Perceived Stress Scale, STAI	R-fMRI (asleep)	Postnatal maternal stress is positively associated with infant negative affect at age 3 months. Peak stress in late pregnancy is associated with increased FC between amygdala and vmPFC.
Rogers et al. [74]	65	1 month	Internalizing symptoms (age 2), BI (age 2)	Infant Toddler Socio-Emotional Assessment	R-fMRI (asleep)	Greater amygdala-dorsal anterior cingulate connectivity was associated with generalized anxiety. Greater amygdala-medial prefrontal cortex connectivity was related to BI.
Salzwedel et al. [98]	223	9 months to 4 years	Toddler anxiety (4 years)	Behavior Assessment System for Children	R-fMRI (asleep)	Growth of amygdala FC with precuneus/cuneus during the second year of life positively correlated with anxiety at age 4 years. Growth of amygdala FC with precuneus/cuneus during the second year of life negatively correlated with anxiety at age 4 years.
Thomas et al. (2019)	62	1 month	Fear	IBQ, ECBQ	R-fMRI (asleep)	Greater newborn amygdala-insula connectivity was associated with greater fear.
DTI						
Graham et al. [84]	34	2 weeks	Maternal anxiety and depression	STAI; Beck Depression Inventory	DTI (asleep)	Maternal anxiety and depression are negatively correlated with infant white matter development in frontal, parietal and temporal regions, as well as the limbic system.
Planalp et al. [83]	103	1–18 months	Infant negative reactivity (fear/distress to novelty) Sadness Anger/distress to limitations	IBQ	DTI (asleep)	White matter microstructure in the left stria terminalis at 1 month of age is associated with infant fear at age 6 and 18 months but not anger or sadness.
Rifkin-Graboi et al. [85]	54	5–17 days	Maternal anxiety and depression Infant internalizing and externalizing behavior	STAI; Edinburgh Prenatal Depression Scale Infant Toddler Socio-Emotional Assessment	DTI (asleep)	Increased maternal anxiety is associated with lower FA in the insula of the infant. White matter microstructure variations do not predict infant internalizing and externalizing behavior.
Delta-Beta Coupling (DBC)						
Anaya et al. (2025)	201	8, 12, 18, 24 months (longitudinal)	Maternal anxiety Negative Affect	Beck Anxiety Inventory IBQ, TBAQ	R-EEG (awake)	High negative affect was associated with delta-beta decoupling (i.e., low DBC). Maternal anxiety was associated with developmental trajectories in DBC that were atypical.
Brooker et al. (2015)	77	6 months	Neuroendocrine reactivity	Lab TAB Cortisol reactivity to laboratory assessment	R-EEG (awake)	High cortisol-reactive infants showed greater DBC in non-fear contexts than during fear eliciting contexts.
Brooker et al. [114]	122	3–5 years	Fear Internalizing	Lab TAB Health and Behavior Questionnaire	R-EEG (awake)	Low observed fear at age 3 was associated with a trajectory of DBC that began low and increased over time. High DBC at age 3 that declined over time was associated with lower internalizing at age 5.
Najjar et al. [110]	90	3 years	Fear	Lab TAB	R-EEG (awake)	Greater DBC was associated with greater social fear.
Phelps et al. [111]	30	4.5 years	Dysregulated fear	Lab TAB	R-EEG (awake)	Toddlers with high levels of dysregulated fear at age 2 years show greater levels of DBC at age 4.5 years compared to toddlers with low levels of fear.
Frontal asymmetry						
Calkins et al. [120]	81	9 months	NR BI	Observed assessments (Kagan reactivity and BI assessments) TBAQ	R-EEG	Greater right frontal asymmetry was associated with NR.
Fox et al. [121]	48	4 years	BI, Social withdrawal	Observed assessments (Kagan BI assessments) Speech task Colorado Temperament Inventory	R-EEG	Children who exhibited greater social withdrawal exhibited greater right frontal activation.
Sacks et al. [166]	323	5 years	Internalizing (maternal and child)	Child: Child Behavior Checklist Mother: STAI	R-EEG	Greater maternal anxiety was associated with greater right frontal asymmetry which in turn. was associated with greater child internalizing at age 5.
Smith and Bell (2009)	48	10 and 24 months	Internalizing	Child Behavior Checklist	R-EEG	Children with stable right frontal EEG asymmetry had greater internalizing symptoms.

*NR* negative reactivity, *BI* behavioral inhibition, *dACC* dorsal anterior cingulate cortex, *DAN* dorsal attentional network, *DTI* diffusion tensor imaging, *EEG* electroencephalography, *FA* fractional anisotropy, *FC* functional connectivity, *FPN* frontoparietal network, *MMR* mismatch response, *PFC* prefrontal cortex, *R-fMRI* resting-state functional magnetic resonance imaging, *SMN* somatomotor network, *T-fMRI* task-based functional magnetic resonance imaging, *vlPFC* ventro-lateral prefrontal cortex, *vmPFC* ventro-medial prefrontal cortex, *IBQ* Infant Behavior Questionnaire, *TBAQ* Toddler Behavioral Assessment Questionnaire, *ECBQ* Early Childhood Behavior Questionnaire, *BIQ* Behavioral Inhibition Questionnaire, *Lab TAB* Laboratory Temperament Assessment Battery, *DBC* Delta-beta Coupling, *STAI* State-Trait Anxiety Inventory.

Similar patterns of rs-FC have also been found in relation to known maternal risk factors for anxiety, including prenatal stress and maternal psychopathology (e.g., [78–82]). However, the direction of these associations does not always parallel studies on temperament. To illustrate, Marr et al. [80] demonstrated that greater prenatal maternal stress, especially in late pregnancy, was associated with heightened within-SN connectivity (i.e., greater amygdala–insula and amygdala–ventromedial prefrontal cortex [PFC] connectivity)—a pattern of rs-FC that had previously been associated with greater negative affectivity from 3–12 months [73]. However, in this sample, elevated late pregnancy stress was associated with lower negative affectivity (a measure that is not specific to fear) at 6 and 12 months. Thus, work on maternal stress and psychopathology often implicates similar networks but remains inconsistent in the direction of these brain-behavior associations. The extent to which this failure to replicate could be in part due to the inconsistency in measurement (negative affectivity is not the same as NR and BI; see Box 1) remains unclear.

While limited, initial evidence suggests that these rs-FC patterns may be paralleled by lower structural connectivity in limbic and prefrontal regions. At 1 month of age, lower fractional anisotropy (FA; an indicator of how organized white matter tracts are in the brain) in the left stria terminalis was associated with higher (and steeper increases in) parent-reported BI [83]. This pattern was specific to fear and not present for other negative emotions (i.e., anger and sadness). The stria terminalis, a white matter tract that includes the bed nucleus and amygdala, develops earlier than other limbic tracts and has also been associated with alterations in adult psychopathology. Higher prenatal anxiety is also associated with lower newborn FA in white matter tracts in the prefrontal regions (including the middle frontal gyrus [84], insula, and dorsolateral PFC [dlPFC] [85]), the middle occipital cortex; [85], the uncinate fasciculus (a white matter tract connecting the amygdala and frontal cortex), posterior cingulate (a key node of the DMN), angular gyrus, and parahippocampus [85]. Similar findings emerge from studies that use maternal depression as the indicator of stress [82, 86–88] and when composite anxiety/depression measures of maternal stress are evaluated in relation to fetal brain connectivity [89]. To date, few studies have measured prenatal maternal stress using questionnaires focused on stressful life events and trauma. This work shows divergent patterns. Humphreys et al. (2020) found that prenatal stress (measured by focusing on stressful life events) was associated with *decreased* newborn amygdala-mPFC functional connectivity and increased newborn amygdala-mPFC structural connectivity. Even so, this is the only study to measure functional and structural connectivity in the same cohort and the sample size was modest, so it remains unclear whether these patterns will be replicable.

When considered as a cluster of brain networks that support attentional control, this work highlights broad consistency among the circuits that are associated with several relevant early-life risk phenotypes (including NR, BI, maternal anxiety, and prenatal stress). However, it is critical to note that these brain networks are varied in their specific roles in supporting the dynamic control of attention. There is a need for additional research to understand the specificity and significance of each rs-FC network associated with early-life risk. It could be that some brain-behavior associations are common across multiple anxiety risk factors (e.g., greater SN connectivity) whereas other patterns are driven by multiple interacting factors.

Delineating why there is inconsistency across findings will be critical for future research. It may be that inconsistency in brain-behavior associations is related (in part) to variability in behavioral phenotype measurement. It is common in the temperament literature to generalize across BI and negative affectivity and in the stress literature to group multiple indicators of stress together to form a composite. Even so, these inconsistencies make it challenging to know whether the variability across studies is driven by meaningful phenotypic differences (see Table 2 for summary of related findings). To date, very few studies have utilized observed measures of NR and BI; thus, there is a need for more research using these measures. We encourage future work to use “big data” and deep phenotyping to resolve these challenges.

**Table 2:** Studies focusing on negative emotionality and other broader temperament dimensions.

Author (year)	Sample size (*N*)	Age	Temperament/psychopathology dimension	Temperament/psychopathology measure	Brain measure	Main findings
Banihashemi et al. (2020)	20	3–9 months	Negative and positive emotionality	Lab TAB	DTI (asleep)	Limbic and interhemispheric structural integrity at age 3 months is negatively correlated to negative emotionality at age 9 months, but not to positive emotionality.
Nevarez-Brewster et al. (2024)	116	1–6 months	Negative emotionality	IBQ Lab TAB	DTI (asleep)	Higher neonatal uncinate fasciculus FA was associated with greater negative emotionality.
Ravi et al. (2023)	74	1 months	Negative affectivity (age 6 mo) Internalizing symptoms (age 18 mo)	Recordings of infant at home (via LENA); IBQ; Child Behavior Checklist	R-fMRI (asleep)	Greater DMN connectivity was associated with lower negative affectivity at 6 months and lower internalizing symptoms at 18 months.
Ravicz et al. (2015)	24	7 months	Surgency/extraversion, negative emotionality, orientating/regulation	IBQ	Emotion processing fNRIS (awake)	Oxyhemoglobin responses to happy face stimuli over the prefrontal cortex are negatively correlated with infant temperament factors.
van der Kant et al. (2018)	37	5–8 months	Negative affect	IBQ	Social processing fNIRS (awake)	Processing of social stimuli activates the right posterior temporal cortex. Infants with high negative affect show a reduced differential response to social vs. non-social stimuli.
Zhang et al. (2025)	39	3–9 months	Negative emotionality, Positive emotionality, Soothability	IBQ	DTI (asleep)	Larger increases in white matter microstructure indices in the uncinate fasciculus, forceps minor and cingulum bundle from 3 to 9 months of age are associated with larger decreases or smaller increases in soothability and positive emotionality.

*DTI* diffusion tensor imaging, *fNIRS* functional near-infrared spectroscopy, *IBQ* Infant Behavior Questionnaire, *Lab TAB* Laboratory Temperament Assessment Battery, *LENA* Language Environment Analysis.

#### Longitudinal changes in rs-FC

A core assumption in our neurodevelopmental models of anxiety is that developmental continuity in phenotypes may be paralleled by continuity in neurobiology [32]. In some ways, an immutable neural marker or brain-based cause would support early identification and explain the similarity in NR, BI, and later anxiety. However, this idea is challenging to reconcile with the profound brain and behavioral changes (and neuroplasticity) that characterize infancy. We know that attentional control [90–97] undergoes critical changes in infancy. This raised the question: to what extent do individual differences in rs-FC remain stable over infancy.

While longitudinal infant MRI research is challenging to conduct (and remains scarce), a few studies have begun to explore rs-FC changes over time and their relation to early-life risk for anxiety. Salzwedel et al. [98] found that increases in amygdala–DMN and amygdala–visual network rs-FC and decreases in amygdala–sensorimotor network rs-FC from ages 1 to 2 years were associated with heightened anxiety at age 4 years [98]. This evidence suggests that changes in infant rs-FC relate to individual differences in anxiety outcomes in early childhood. While promising, less than 30 infants in this study provided complete data across all three longitudinal imaging timepoints (i.e., age 0, 1-, and 2-years), raising questions about the reliability of the observed patterns and extension to behavioral phenotypes of risk.

To expand on this work, our group conducted the first investigation of infant rs-FC change in relation to parent-reported BI using the Baby Connectome Project (BCP) dataset. BCP is unique in that it utilized a cross-sequential study design—recruiting cohorts that differed in terms of age at study onset and then longitudinally scanning them in predetermined intervals. This study design allows for rich longitudinal modeling, capturing change across the first three years of life with unprecedented precision (covering nearly all months from 0 to 36). By characterizing attentional-related network change across 180 infants (396 independent imaging sessions) we showed that less change in the connectivity between DAN-FPN is associated with greater parent-reported BI at age 2 years [99]. In follow-up analyses, we utilized all available imaging and BI data to relate co-trajectories of parent-reported BI and rs-FC. These results replicated our focal analyses and demonstrated that smaller changes in DAN-FPN rs-FC related to increasing BI over time [99]. These findings are consistent with theories suggesting that altered development of networks that support attentional control underlie the maintenance of fear over time [100]. Patterns of less change over time in those with early-life risk phenotypes have also been observed in the development of the amygdala in middle childhood [101], suggesting that slowed development within attention networks could also be a defining feature of risk. While the functional significance of less change in DAN-FPN rs-FC remains unknown, decreases in rs-FC have previously been associated with greater functional independence or specialization of networks [17]. In early infancy, high rs-FC between networks suggests highly coordinated activity across both networks (i.e., less network specialization). Over time between network rs-FC decreases for several networks which is thought to reflect increases in network specialization. Building from this work, our findings could indicate that infants who develop BI show reduced network specialization or a more tightly coupled DAN-FPN circuit which (over time) impacts attention switching. However, this interpretation is speculative and at present we don’t know how these rs-FC changes impact task-based information processing.

The longitudinal rs-fMRI work reviewed here demonstrates that when we evaluate within-subject changes in rs-FC over time, different associations emerge between rs-FC associated with anxiety in preschoolers and parent-reported BI. Further exploration of whether slowed functional specialization of DAN-FPN relates to anxiety outcomes will be critical for illuminating the clinical significance of the BI findings, as will studying larger samples of infants with anxiety outcome data.

### Resting state EEG (rs-EEG)

Although fMRI can provide critical insight into the brain regions that are being engaged, due to limitations of data collection in infancy, it primarily informs our understanding of functional connectivity during sleep. Resting state EEG (rs-EEG) is an inexpensive tool that is both relatively well-tolerated by *awake* infants and can be easily obtained in a variety of contexts (including the clinic/hospital, homes, or in a laboratory setting). To date, two measures of rs-EEG remain prominent in the infant anxiety risk literature: (1) delta-beta coupling and (2) frontal asymmetry. In the section that follows, we outline each of these measures and review evidence that these rs-EEG measures relate to early-life behavioral markers of risk.

#### Delta-beta coupling (DBC)

Delta-beta coupling (DBC) refers to temporal correlations between power (i.e., amount of neural activity) in the delta (0.5–2 Hz) and beta (11–18 Hz) frequency bands. Delta power is thought to index bottom-up processes (e.g., salience processing), whereas beta power is thought to index top-down control mechanisms (e.g., control of subcortical systems). Some have argued that greater DBC may reflect an exaggerated neural regulation mechanism [102] (speculatively, amygdala-mPFC circuitry). In adults and children, greater DBC is associated with anxiety [103–108] and BI [105, 109]. A growing body of work suggests that similar patterns may be present earlier in development. Three-year-olds high in BI show greater DBC [110]. Furthermore, relative to non-dysregulated toddlers, toddlers with dysregulated fear (see Box 1) exhibited greater DBC at age 4 [111]. Relative to low reactive infants, infants with high stress reactivity (a behavioral classification with similarities to negative emotionality) exhibited greater DBC [112]. These findings suggest that high DBC could be an early-life correlate of temperamental risk for anxiety.

To date, two studies have evaluated longitudinal changes in DBC over infancy and toddlerhood. These studies showed that infant frontal DBC is stable over the first few months of life and that DBC may be influenced by maternal anxiety and infant negative affect [113]. Although in this case, associations with infant negative affect were in the opposite direction as prior work (i.e., negative affect was associated with decoupling). In a study of toddlers, low BI was associated with a trajectory of DBC that began low and increased over time [114]. Although in this study, they additionally found that high DBC at age 3 that declined over time was associated with lower levels of internalizing at age 5. Together, these longitudinal studies suggest that those at highest risk may exhibit lower DBC, a pattern that is inconsistent with cross-sectional work. Future research should explore why this could be and should consider the value in enriching samples with mothers with anxiety disorders or BI children.

#### Frontal EEG asymmetry

In addition to measuring temporal correlations between electrical activity at the scalp, researchers have also examined the relative balance of electrical activity in the left and right hemispheres of the frontal cortex, a measure called “frontal asymmetry.” Greater right hemisphere activation (measured via EEG power) relative to left hemisphere (i.e., right frontal asymmetry) is thought to index higher avoidance/withdrawal propensity [115]. The mechanisms underlying avoidance sensitivity remain underspecified (particularly within the spatial domain). Even so, in line with data on children [116, 117] and adults [118, 119], greater right frontal asymmetry in infancy is associated with NR [120], BI [121–123], and internalizing [124]. However, this pattern is not specific to these temperaments; similar patterns have been observed in infants who experience early deprivation [125] and among those with depressed mothers [126].

While limited, a small number of studies have evaluated infant frontal asymmetry over time. These data overwhelmingly suggest that resting frontal asymmetry measures are not stable over time—leading some to suspect that it is not a stable trait maker but instead indicative of the infant’s current state [122, 123, 127]. Even so, one study has shown that stability in BI over the first four years of life was associated with right frontal EEG asymmetry at two distinct timepoints in infancy (9 and 48 months) [122]. Longitudinal modeling of right frontal EEG asymmetry and BI from 10 to 48 months indicates that BI may precede this neural marker [123]. Nevertheless, two studies have found longitudinal associations suggesting that right EEG asymmetry tracks with child outcomes. These studies find that stable trajectories of right frontal EEG asymmetry [124] and increases in right EEG asymmetry [125] in infancy/toddlerhood predict internalizing symptoms at age 4. Similar effects persist into early childhood and across other temperament measures (e.g., negative emotionality has also been associated with reductions in left frontal asymmetry from preschool to age 6 [128]).

Together, this work provides evidence that right frontal EEG asymmetry may be correlated with NR, BI, and internalizing symptoms beginning in infancy and early childhood. Furthermore, while (on average) frontal EEG asymmetry lacks reliability over time, stable and increasing right frontal EEG asymmetry have been associated with internalizing symptoms. This could indicate that the trajectory of right frontal EEG asymmetry over development is less varied among the most at-risk individuals. In comparison to many other neural measures reviewed, the brain-behavior association observed here is relatively consistent in direction across measurement differences and study type (e.g., cross-sectional vs. longitudinal). Additional work is needed to understand the functional significance of right frontal asymmetry. This could include exploring the relation between right frontal EEG asymmetry and other measures of the brain at rest and/or task processing.

## Infant stimulus processing: salience and emotion processing

While studies of the brain at rest have provided key data about how task-*independent* neural processing relates to anxiety, far more work has been done studying the anxious adult and child brain while performing a task. Although challenging, studying how the infant brain engages during attention-demanding tasks has also shed key insight into early-life risk for anxiety. In this section, we overview what we have learned by investigating differences in how the infant brain responds to salient and emotional stimuli.

### The auditory oddball

**Fig. 2:**
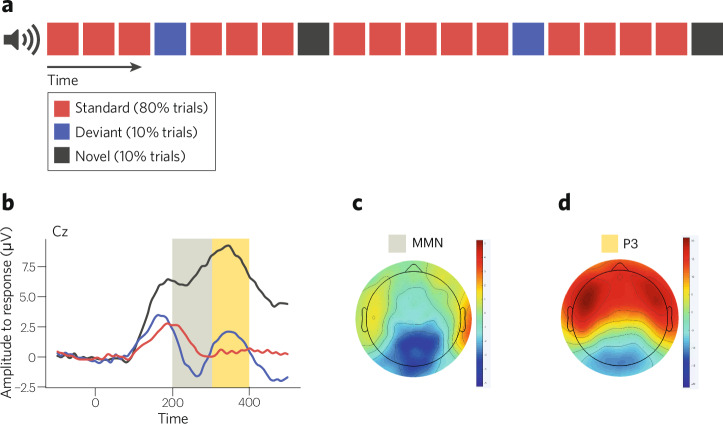
Three-stimulus auditory oddball paradigm and related event-related potentials. **a** Auditory oddball stimulus presentation paradigm. The subject hears a series of “beep” sounds consisting of two distinct tones: the “standard” tone (a 1 kHz frequency) is presented at random intervals for 80% of the trials; the “deviant” tone (a 1.5 kHz frequency) is presented at random intervals for 10% of the trials. For the remaining 10% of the trials novel sounds are presented, such as a cow mooing. **b** Grand average waveform from a sample of 5-month-olds (*N* = 55). The gray box highlights the time window when we evaluate the “mismatch negativity” (MMN) response (Deviant— Standard). The beige box highlights the novelty P3 neural response (Novel—Standard). Scalp topographic plots showing MMN (**c**) and novelty P3 (**d**) across electrode sites (hot colors indicate positive ERP values; cool colors indicate more negative ERP values).

Infancy is characterized by a limited motor repertoire and (initially limited but) rapidly developing perceptual abilities. Unlike adults, infants often cannot follow instructions. These features of infancy can make it challenging to study the infant brain during stimulus processing. As such, researchers often use shorter, passive tasks to ensure infant compliance and engagement. Since adults are typically expected to produce a behavioral response to demonstrate compliance and engagement, it is difficult to compare infant task-elicited neural responses to that of adults. Still, one task that has been particularly fruitful for examining attention across adults and infants is the “auditory oddball.” The auditory oddball is a passive listening task that can be collected alongside recordings of brain activity. During a three-stimulus auditory oddball task, participants hear a predictable stream of identical sounds (i.e., “*Standard”* sounds, such as 1000 Hz tones) interposed with infrequent but unvarying sounds (i.e., “*Deviant”*, such as 1500 Hz tones; [129, 130] and/or infrequent, new and varying sounds (i.e., “*Novel”*, such as a horn blaring, cow mooing, etc.; see Fig. 2). This task allows us to evaluate how the brain attends to changes in the sensory environment (i.e., neural response to the *Deviant* condition) and to salient stimuli (i.e., neural response to the *Novel* condition; [131–133]).

EEG recorded during a three-stimulus auditory oddball allows us to extract two time-locked neural signatures (i.e., event-related potentials [ERPs]) over fronto-central sites: “mismatch negativity” (MMN) and novelty P300 (see Fig. 2; also referred to as the novelty P3). The MMN is a neural response that peaks between 100 and 250 ms after a *Deviant* stimulus. In contrast, the novelty P300 is a response that peaks around 300 ms after a *Novel* stimulus. These responses can be measured within the first days of life [134–139], with no significant differences observed between responses recorded during wakefulness and those recorded during sleep [140, 141].

Infants with temperamental risk for anxiety exhibit distinct neural responses towards deviance when measured using the auditory oddball EEG and fMRI tasks. To illustrate, Marshall et al. [142] found that infants who exhibited NR at 4 months had a greater MMN response at 9 months of age [142]. These findings provide neurobiological evidence supporting the idea that at-risk infants may have heightened sensitivity to the sensory environment. These infant EEG findings bear striking resemblance to recent fMRI findings, which demonstrated that newborns who exhibited larger (earlier) PFC responses to *Deviant* sounds had greater BI one year later [143]. Research has also shown that relative to newborns of non-anxious mothers, newborns of anxious mothers exhibit larger neural responses to *Deviant* sounds in the SN (including insula, ventrolateral PFC, and anterior cingulate cortex [ACC; [144]]). Together, these studies suggest that early-life risk is associated with greater processing of unexpected sensory changes in brain regions responsible for detecting salient cues and regulating behavior. The convergence across imaging modalities and with research done in older samples [145, 146] is highly promising and suggests that rapid processing of sensory deviance may provide clues about who is most at risk for anxiety.

This work raises new questions about how heightened neural responsivity to sensory changes may emerge in the context of reduced DAN-FPN rs-FC. Task-elicited activity is thought to guide network connectivity (i.e., the principle that “neurons that fire together, wire together”). However, in the studies reviewed to date, we’ve highlighted a pattern that seems contradictory: at-risk infants show both heightened neural sensitivity to the sensory environment and reduced DAN-FPN rs-FC over time. Rather than suggesting that greater attention to the environment doesn’t change rs-FC, speculatively, it could be that heightened sensitivity to the environment shapes learning such that your attentional control networks become less efficient at shifting your attention away from the change. Evaluating this possibility could be achieved using experimental manipulations that target sensory learning.

### Emotion processing

In addition to heightened sensitivity to changes in the sensory environment, anxious children attend to [147–149] and process [150–152] emotion and perceived threat differently than non-anxious children. Infants are highly tuned to emotion; by 4 months, infants discriminate between emotional expressions [153] and by 24 months use others’ emotional reactions to inform their own behavior [154]. Researchers have begun exploring how differences in infant neural responses to emotional stimuli (e.g., fearful faces) relate to early-life risk for anxiety. To illustrate, using functional near-infrared spectroscopy (fNIRS), Kelsey et al. [155] demonstrated that 7-month-old infants who exhibited greater right frontal activity (over the dlPFC) to happy and fearful faces had an increased likelihood of developing an anxiety diagnosis at age 5 [155]. This suggests that at-risk infants may also engage the right dlPFC more than the left dlPFC during stimulus processing as well as at “rest”. Although the opposite pattern (i.e., greater left frontal EEG activation during emotion processing is associated with parent-reported fear at 8 months) has also been reported [156].

Maternal anxiety and stress also shape infants’ neural processing of emotion. To date, two infant studies show a positive association between maternal anxiety and the strength of infant neural responses (as indicated by ERP amplitudes) to fearful stimuli [157, 158]. Bowman et al. (2021) showed that an enhanced Nc amplitude was associated with greater maternal anxiety. This association was selective; it was not present for emotional expressions of anger or happiness. Large Nc amplitudes are associated with attention orienting and emotional arousal, suggesting that infants at risk for anxiety may exhibit elevated attention orienting or arousal to fear. Similarly, Otte et al. [157] found that 9-month-old infants exposed prenatally to higher maternal anxiety exhibited a larger P350 response (thought to be an infant precursor to the P300; [136]) to fearful vocalizations. The P300 is also associated with attention orienting (particularly involuntary shifts to salient changes in the environment). Thus, both studies suggest that greater exposure to maternal anxiety (pre- and postnatally) is associated with larger neural orienting responses to fear in infancy. Although this association may not be unique to maternal anxiety, infants who are exposed to high levels of parental conflict show stronger neural responses to angry (relative to neutral) speech in brain regions associated with the SN [159]—potentially indicating that the infant brain is learning from the cues most present in its environment. Together, this work illustrates that the infant brain is highly sensitive to its environment and exhibits larger responses to the stimuli that are most readily available.

Taken together, these data support the hypothesis that those infants at greatest risk for developing anxiety have a neurobiological predisposition to process information differently from those who are not at risk—potentially from birth. Specifically, by studying moment-to-moment fluctuations in stimulus processing using multiple imaging modalities, research has illustrated that at-risk infants exhibit differences in the rapid components of stimulus processing, particularly in the context of unexpected stimulus changes and fearful stimuli. It could be that a neurobiological predisposition shapes how infants attend to, process, and learn from their social environment. In considering the implications of this claim, we urge researchers to consider the unique experiences and challenges that the infant brain faces. Infant brains are not immature versions of the adult brain. Infant brains are wired to develop attachments to their caregivers and learn about their environment. While we know that over time, at the group level, these learning mechanisms can facilitate the acquisition of complex skills (e.g., language), we know far less about how individual differences in these mechanisms shape individual differences in behavior and child outcomes. This raises new questions about how early exposure to anxiety shapes developing neural systems that support emotion and association/contingency learning. This is an active area of research in the adolescent anxiety literature [160, 161], that has not yet been explored in infancy. By expanding research on how at-risk infants learn contingencies in their social environment, we may identify key neural targets for intervention. Overall, the complementary insights gained from EEG, fMRI, and fNIRS provide a strong foundation for future research while raising important questions about how to compare findings across modalities.

## Pathways from temperamental risk to anxiety

When we study groups of infants at risk, we do so knowing that not all infants with risk phenotypes (i.e., NR, BI) ultimately develop anxiety. For this reason, it is critical to also study individual trajectories to anxiety. In this section, we review recent research that is beginning to unravel how these neurobiological profiles contribute to the risk trajectories from infancy to adolescence. The first section reviews research showing that neural measures collected at rest explain pathways from early-life risk to anxiety. The second section builds on work demonstrating that those infants that exhibit both BI and heightened attention to subtle environmental changes are most at risk for developing anxiety as adolescents [162–165]. In doing so, we consider infant neurobiology as a key moderator of risk. We conclude with a discussion on the value of considering neural measures as both mediators and moderators of early-life risk pathways.

### Mechanistic pathways: the brain at rest

The search for pathways to anxiety has led many to speculate that the brain might explain associations between early-life markers of risk and the development of anxiety later in life. If this were the case, this would be strong evidence that changes in the brain could reduce child anxiety later in life, providing neural targets for early intervention. While this could have enormous clinical potential, to date, very little evidence suggests that neural measures of the brain at rest explain relations between risk and later symptoms. Indeed, no studies of the infant brain have illustrated this effect. However, one study of preschool children has found that right frontal alpha asymmetry at age 5 mediates the relation between maternal anxiety and child internalizing symptoms at age 7 [166]. This suggests that right frontal asymmetry could be a mechanism that explains the transmission of anxiety from parents to their children (i.e., intergenerational transmission of internalizing disorders). Given the recency of this discovery, there are still many open questions about whether the same could be true earlier in development and how to modify right frontal asymmetry. Even so, this work highlights the possibility that neural indicators captured during rest could reveal neurobiological mechanisms of risk transmission.

### Information processing shapes anxiety outcomes

**Fig. 3:**
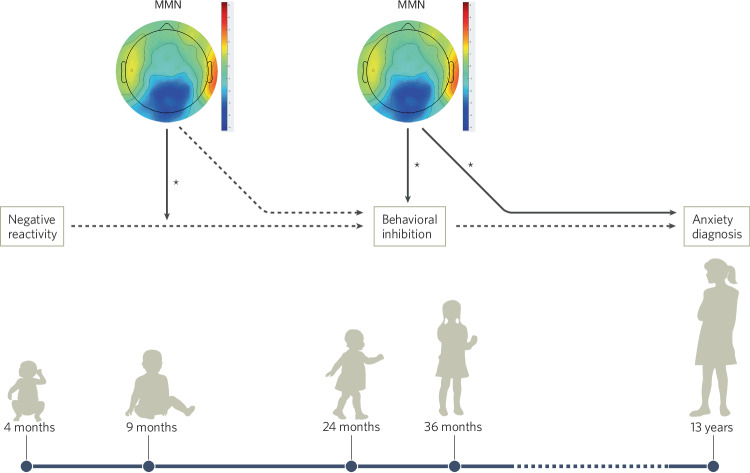
Structural equation modeling results summarized (Xing, Kanel, et al., under review). Results indicated that MMN at 9 months moderates the relation between NR and BI and the MMN at 36 months moderates the relation between BI and anxiety. MMN at 36 months was also significantly associated with anxiety.

Our recent work shows that beginning in infancy neural processing during attention-demanding tasks influences risk trajectories. Leveraging data from the Temperament Over Time study, a large cohort study that began when infants were 4 months old and continued for more than 15 years to evaluate trajectories of social-emotional development, our group explored the *moderating* impact of the infant MMN and P300 on risk trajectories to anxiety. This study included assessments of NR (observational assessment), BI (observational assessment and parent report), and anxiety symptoms (self- and parent-reports and clinical interviews) during adolescence. Infants also took part in a three-stimulus auditory oddball task with simultaneous EEG recording at 9 months and 3 years of age. Using structural equation modeling and data from 291 participants, we evaluated whether infant neural processing of changes in the environment or salience (measured as MMN or novelty P300, respectively) moderated the paths from NR to BI, and from BI to anxiety. We found that infants with higher NR (at 4 months) and a larger infant MMN (at 9 months) exhibited greater BI (at 2–3 years). Furthermore, toddlers with greater BI (at 2–3 years), and who exhibited a larger MMN (at 3 years), exhibited greater anxiety in adolescence (see Fig. 3). Thus, enhanced neural processing of unexpected (i.e., *Deviant*) stimuli modulated the maintenance of temperamental risk from infancy to toddlerhood, as well as the pathway from temperament risk to anxiety in adolescence. This effect was selective to the MMN; no significant effects emerged when we tested the moderating impact of the novelty P300. A pattern that diverges from prior studies of the adolescent brain [164]. Together, this study highlights that the MMN in conjunction with behavioral markers of risk could indicate which infants are more likely to exhibit anxiety later in life—providing promising evidence that neurobiology could support early identification efforts.

Attention to emotional faces also modulates temperamental risk trajectories. Rayson et al. [167] investigated how elevated neural responsivity to faces with different emotional expressions (happy, angry, neutral) influences trajectories from BI to anxiety. Results showed that preschoolers who had a larger N2 response to angry faces and greater BI exhibited the highest anxious symptoms [167]. The N2 is an attentional ERP component that, in adults, is generated by the ACC and the orbitofrontal cortex [168] and is associated with attentional control. These findings converge with studies in older children showing that larger N2 amplitudes moderated the relationship between shyness and social anxiety [169]. This work demonstrates that anxiety risk is also amplified in individuals who show enhanced neural processing of threatening stimuli (i.e., angry faces) and behavioral markers of risk. These findings reiterate that measuring neurobiological processes in combination with behavior could enhance early identification efforts.

Together, this work provides some of the first evidence that the brain may provide clues about which infants are at greatest risk for developing anxiety. In doing so, these studies illustrate the value in translating the group-level neurobehavioral profiles into hypothesis-driven longitudinal models. This work exemplifies how studying the infant brain adds value to our understanding of risk trajectories. By studying the infant and toddler brain alongside behavior, this work was able to leverage variability across individuals and across time to inform the developmental pathophysiology of anxiety.

## Future research directions

As early as infancy, we have been able to document early neurobehavioral profiles of risk. This work overwhelmingly suggests that early-life risk is characterized by differences in neural circuits that support attention and control. Even so, our understanding of the functional specificity that this circuitry has on risk profiles remains limited. This section highlights areas for additional translational research (see Table 3 for summary).

**Table 3:** Considerations for future work.

Study design type	Value added	Additional key design considerations	Key questions of interest	Example
Longitudinal designs	Facilitates tracking risk trajectories prior to the onset of symptoms	Measure temperament using observed assessments of NR and BI	What factors support the trajectory from NR to BI and BI to anxiety?	Filippi et al. [99], Schwarzlose et al. [143], Planalp et al. (2022)
	Facilitates the identification of developmental transitions	Obtain measures of infant attention	Is the trajectory from NR to BI related to specific attention patterns?	
	Facilitates tracking risk from genetic influences and prenatal environment	Include measurements of maternal factors beginning in pregnancy Enriching samples for maternal anxiety (or other maternal factors associated with risk)	What prenatal factors precede NR? What prenatal factors support the trajectory from NR to BI? Do patterns observed in community samples translate to those with genetic risk?	Sylvester et al. [144]
	In the absence of experimental manipulation, facilitates investigations into how context may influence risk trajectories	Observe parent-child interactions	Do any features of parent-child interactions moderate infant risk trajectories or precede NR or BI? Does caregiving context impact infant brain development?	
Big data studies (e.g., mega-analyses or meta-analyses)	Allow us to evaluate how study-to-study variability may be impacting brain-behavior associations reported in the literature	Compare HBCD data to patterns identified in the extant literature	Are there reliable neurobiological profiles of risk?	
			How generalizable are the effects observed in the literature?	
Multi-modal studies	Enhance translation across modalities and support hypothesis generation	Resting-state measures vs. task-evoked responses	How do EEG signatures relate to fMRI signatures of risk?	Kanel et al. [184]
		Temporal and spatial resolution across each modality impacts interpretation		
Experimental studies	Facilitate causal explanations	Manipulations of the caregiving environment	Do changes in caregiver behavior support changes in ERP responses to emotion or unexpected stimuli?	
		Manipulations of infant attention/contingency learning paradigms	Does infant attention training result in changes in attention-related brain networks?	

### Prenatal period

**Fig. 4:**
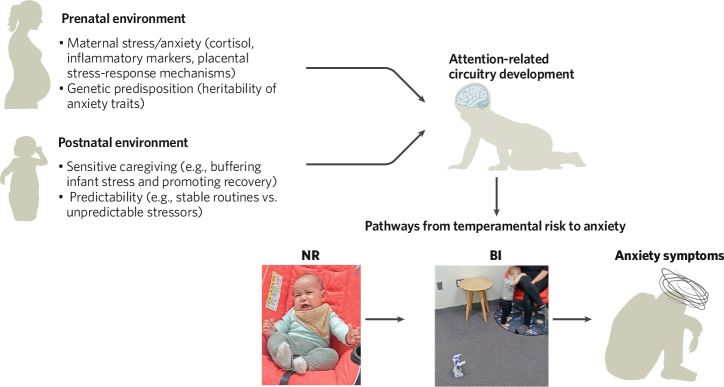
Conceptual model illustrating how pre- and postnatal environment may shape attention-related neural circuitry development, which in turn shapes trajectories of risk. Trajectories of risk are depicted as the path from negative reactivity to behavioral inhibition to anxiety. Authors affirm that they obtained informed consent for publication of the images.

The observation that newborn attention-related brain networks exhibit differences that precede the expression of BI points to potential neural programming modifications beginning in the fetal period. Growing evidence suggests that prenatal stress could impact the fetal environment via utero-placental mechanisms [170] in ways that subsequently shape brain development [89, 171]. It has been speculated that, in infancy, the neural circuits that support attention may be especially vulnerable to environmental cues of stress, uncertainty or threat, which over time could influence the development of BI [100]. However, at present, neurodevelopmental models of anxiety are underspecified in terms of how contextual (from gestation or infancy) and/or genetic factors impact neurobiology and trajectories of risk. To support theoretical refinement (see Fig. 4), we need additional data on maternal biology, fetal exposure to stress, and genetics. New studies should consider enriching for maternal stress, anxiety, or environmental stressors (e.g., trauma) and conducting deep phenotyping of the mother (over the course of pregnancy) and child over the first year of life. This work could advance our understanding of brain development and delineate pathways for prenatal transmission of risk.

### Big data

While this review highlights that attention-related neural circuitry is linked to risk, at present, it is difficult to ascertain how specific this neurobiological profile may be. In part, this is a challenge related to small samples and enormous variability in the measurement of brain and behavior. To date, no replications have been performed, and data do not converge on specific brain regions that could be targeted [172]. Rather, nodes across several different attention networks have been implicated in early-life risk. The factors accounting for study-to-study variability in findings remain unclear. Studies vary in measurements of fearful temperament (e.g., parent report, observed behavior), age of behavioral assessment, demographics, inclusion criteria (e.g., inclusion of preterm infants), and imaging analysis methods. Thus, while some neural markers are consistently implicated, it remains unclear whether any are reliable precursors to early-life risk or anxiety. Mega-analysis, the process of pooling and harmonizing raw data from multiple independent studies/sites and processing the data centrally using a single computing environment and analysis framework, is one method that could be used to understand which findings are most reproducible. This approach improves statistical power by obviating differences in processing pipelines and accounting for the confounding effects of sample demographics. Mega-analysis has already enhanced reproducibility in other areas of psychiatry [173, 174]. However, in the subfield of infant MRI studies, this approach remains nascent [175–178].

Large-scale longitudinal neuroimaging efforts, such as the HEALthy Brain and Child Development (HBCD) study [179–181], hold tremendous promise for advancing reproducibility efforts. HBCD’s large (*N* ~ 7000), open-access dataset is comprised of rich measures of maternal experience beginning in pregnancy (e.g., stress and trauma), measures of the home environment, and includes multiple measures of infant temperament (both observed [i.e., Lab TAB] and parent-report [e.g., Patient-Reported Outcomes Measurement Information System [PROMIS] [182]) and the developing brain (including longitudinal rs-fMRI, rs-EEG, and a two-stimulus auditory oddball task with EEG). These data will allow us to explore the reliability of brain-behavior associations, facilitate research on the specificity of infant neurobehavioral profiles of risk, and advance research on longitudinal brain change in at-risk infants. Future research must strike a balance between large-scale longitudinal studies with small-scale, high-resolution studies to achieve robust, generalizable, and mechanistically informative findings [183]. Integrating the strengths of both approaches will enable discoveries that are not only scientifically robust but also applicable to individual-level clinical contexts.

### Multimodal imaging

Throughout this review, we’ve highlighted several neural measures that relate to early-life risk for anxiety. While we’ve classified these measures based on whether they were obtained from the brain at rest or during stimulus processing, within each section we describe specific neural responses across multiple modalities (e.g., EEG, fMRI, diffusion-weighted MRI, fNIRS). These data provide complementary information about neural processing. However, at present, it remains unclear how these different measures of the brain relate to one another. Despite broad agreement that task-elicited activity guides network connectivity (i.e., the principle that “neurons that fire together, wire together”), to date only one study has explored how variability in task-evoked responses relates to rs-FC patterns in infancy. This work showed that greater rs-FC between the DAN and sensorimotor network is positively associated with MMN amplitude [184]. Expansions of this kind of work to other relevant task-evoked neural responses or evaluating how multiple measures of resting-state (e.g., EEG vs. fMRI) relate to one another are needed. Advancements in this area could come from big datasets (e.g., HBCD), existing data, or the development of new simultaneous multimodal studies (e.g., obtaining simultaneous EEG and fNIRS). Developments in this area of research could be particularly valuable for hypothesis generation.

### Context: caregiving and home environment

To date, we have not yet disentangled how the brain-behavior associations observed are affected by caregiving context. This information is critical because caregiver behavior impacts infant stress reactivity [185] and is linked to the maintenance of BI [186] and anxiety in children [187, 188]. Unlike other developmental stages, infancy is characterized by complete dependence on the caregiver. Animal model data provide compelling evidence that the infant brain is wired in a way that maximally leverages input provided by caregivers [189]. Thus, infancy may be a time when caregiver behavior has the greatest impact on the developing brain and the greatest potential for supporting long-term resilience.

To date, very few studies have examined how individual differences in the infant brain relate to parent-child interactions [110]. One such study found that infants with greater SN connectivity exhibit less stress recovery during a still-face reunion episode at 3 and 6 months [190]. This work provides preliminary evidence that how infants engage with caregivers is linked to individual differences in their neurobiology. Research has also begun to show that sensitive responding to infant cues impacts infant neural responsivity [191] and mother-infant brain synchrony [192]. However, it remains unclear whether and how these findings could impact anxiety outcomes. We urge researchers to consider both longitudinal extensions (to evaluate clinical impact) and experimental extensions (e.g., examining how sensitive responding impacts moment-to-moment changes in infant/toddler brain activity; to support causal inferences). Together, this work would set the stage for targeted intervention studies that examine how behavioral modifications impact the infant brain and later outcomes.

To conclude, infancy, a developmental stage rarely emphasized in clinical literature and characterized by profound neural plasticity, has shed important insights into the developmental pathophysiology of anxiety. Throughout this review, we highlight how developmental scientists have leveraged the variability in infant behavior and the brain to understand the mechanisms that underlie trajectories from early-life temperaments to anxiety outcomes. In doing so, this work has expanded on existing neurodevelopmental models of anxiety and illuminated a neurobehavioral profile of early-life risk for anxiety, which involves both behavioral avoidance of novelty and neural processes that support various dimensions of attentional control. Future work is still needed to understand the timing and specificity of these effects and how environmental factors shape developmental trajectories. Even so, we are optimistic about how ‘big data,’ longitudinal studies, and multimodal approaches will further enhance our understanding of early-life risk for anxiety. This work has and will continue to provide novel insights for early identification and intervention efforts.
